# Cross-regulation of amino acid synthesis and anaerobic electron transfer by MetR-mediated methionine signaling

**DOI:** 10.1128/jb.00181-26

**Published:** 2026-06-22

**Authors:** Hisae Mogi, Keisuke Tomita, Atsumi Hirose, Erika Yoshino, Takuya Kasai, Atsushi Kouzuma, Kazuya Watanabe

**Affiliations:** 1 School of Life Sciences, Tokyo University of Pharmacy and Life Sciences 13115, Hachioji, Tokyo, Japan; University of Notre Dame, Notre Dame, Indiana, USA

**Keywords:** *Shewanella*, methionine, extracellular electron transfer, transcriptome

## Abstract

Microorganisms living in nutrient- and energy-limited environments must finely coordinate anabolic and catabolic pathways. However, the molecular mechanisms underlying this balance remain poorly understood. Here, we report a mechanism cross-regulating amino acid biosynthesis and anaerobic respiration in bacterial cells. Using the model bacterium *Shewanella oneidensis* MR-1, we demonstrated that methionine activates extracellular electron transfer activity at submillimolar concentrations. This regulation is mediated by the transcription factor MetR, a canonical regulator of methionine biosynthesis. Under methionine-limited conditions, MR-1 upregulates methionine biosynthesis genes while repressing genes involved in extracellular electron transfer (metal reduction) and other anaerobic respiratory processes. Conversely, methionine availability relieves this repression, enhancing extracellular electron transfer. Furthermore, the MetR-mediated regulation of extracellular electron transfer was observed in *Aeromonas hydrophila*. These findings reveal a novel physiological role for MetR, suggesting its involvement in the coordinated energy distribution between anabolic and catabolic pathways.

**IMPORTANCE:**

This study identifies a novel regulatory link between methionine metabolism and anaerobic respiration. We show that *Shewanella oneidensis* MR-1 uses MetR to repress anaerobic respiratory pathways while activating methionine synthesis. This suggests a resource allocation strategy where bacterial cells prioritize the synthesis of “expensive” amino acids like methionine over the synthesis of respiratory machinery in amino acid-limited environments. Upon methionine availability, this suppression is lifted, boosting anaerobic electron transfer and energy production. Understanding this cross-regulation provides new insights into bacterial survival strategies and offers genetic targets for optimizing microbial electrochemical technologies, such as microbial fuel cells, where maximizing the extracellular electron transfer rate is critical.

## INTRODUCTION

Balancing anabolic and catabolic metabolism is essential for the survival of organisms, especially microorganisms inhabiting nutrient-poor or energy-limited environments (1, 2). These microorganisms must efficiently allocate energy obtained from catabolism (e.g., respiration) to the synthesis of cellular components (e.g., amino acids and proteins). While specific regulators for individual pathways are well characterized (3), the mechanisms underlying the interactive regulation of anabolism and catabolism remain largely unexplored.

*Shewanella oneidensis* MR-1, a facultative anaerobic gammaproteobacterium (4), can utilize various anaerobic electron acceptors, such as fumarate, nitrate, dimethyl sulfoxide (DMSO), trimethylamine *N*-oxide (TMAO), and metal oxides (5). Due to its ease of culture and genetic manipulation, as well as its diverse respiratory activities, *S. oneidensis* MR-1 serves as a model organism for studying how bacteria adapt to redox-stratified environments with limited nutrient and energy availability (6). Moreover, MR-1 is extensively studied as a host for microbial electrochemical technologies (METs), such as microbial fuel cells and electrofermentation systems, because it can link intracellular redox reactions to electrodes through the extracellular electron transfer (EET) pathway (7, 8). Understanding how MR-1 regulates anaerobic respiration in response to environmental cues is critical for advancing our knowledge of microbial survival strategies and optimizing its applications in METs.

MR-1 utilizes the cyclic AMP receptor protein (CRP) to activate the expression of many anaerobic respiratory genes, including those for EET (*omcA* and *mtrCAB*) and DMSO reduction (*dmsEFABGH*) (9, 10). CRP binds directly to the upstream regions of *omcA* and *mtrCAB* and activates their transcription upon receiving the intracellular second messenger cAMP (10). However, the specific environmental stimuli that trigger the expression of CRP-regulated genes remain unidentified. Previously, we demonstrated that MR-1 downregulates the expression of amino acid biosynthesis pathway components under anaerobic, electron acceptor-limited conditions, thus requiring an external supply of amino acids for fermentative growth in defined media lacking terminal electron acceptors (11). This finding suggests the presence of unknown regulatory crosstalk between the amino acid biosynthesis and anaerobic respiration pathways in MR-1.

In this study, we hypothesized that amino acid availability affects the catabolic (respiratory) activity of MR-1 through an unknown regulatory mechanism. Given that MR-1 can use an electrode as an electron acceptor for respiration, its respiratory activity (i.e., electron transfer rate) can be readily quantified in real time by monitoring current generation in an electrochemical cell (EC) (12). Therefore, to test our hypothesis, we investigated the effect of external amino acid supplementation in defined media on current generation by MR-1.

## RESULTS

### Effects of amino acids on the EET activity of MR-1

**Fig 1:**
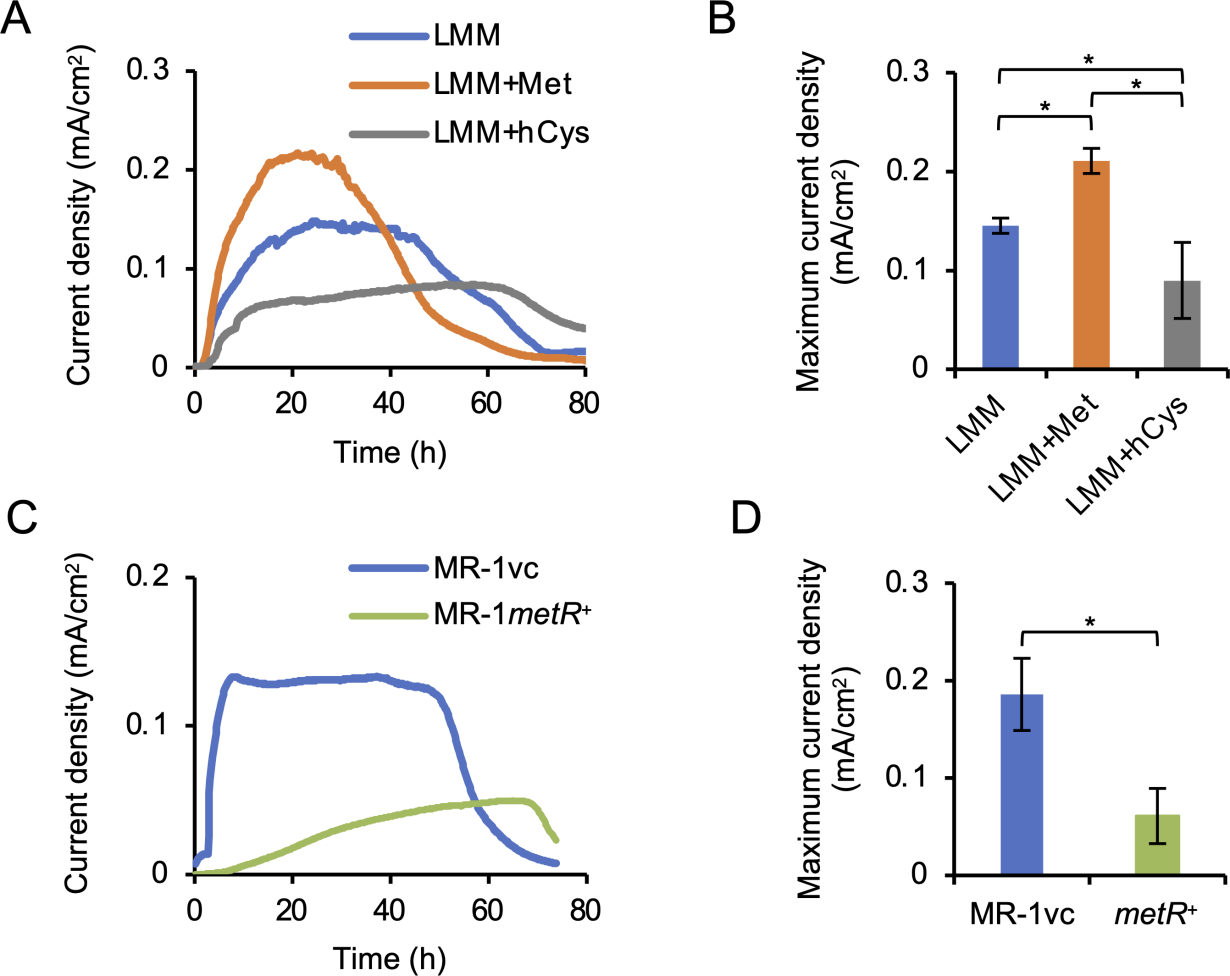
Effects of Met, hCys, and *metR* expression on current generation by *Shewanella oneidensis* MR-1. (**A**) Representative current density vs time curves and (**B**) maximum current densities for wild-type MR-1 in ECs containing LMM supplemented with 130 µM Met or hCys. (**C**) Representative current density vs time curves and (**D**) maximum current densities for MR-1vc and MR-1*metR ^+^* in ECs containing LMM. ECs were operated at a working electrode potential of +0.4 V (vs the standard hydrogen electrode). In panels **B and D**, the bars and error bars represent the means and standard deviations, respectively (*n* = 3 biological replicates). Asterisks indicate statistically significant differences (*P* < 0.05; one-way analysis of variance followed by Tukey’s honestly significant difference test in panel B and Student’s *t*-test in panel **D**).

To explore the amino acids that influence the EET activity of MR-1, we assessed the current generation by this strain in ECs filled with lactate minimal medium (LMM, containing 10 mM lactate as the carbon and energy source) supplemented with 1 of the 20 standard amino acids (130 µM each) (Fig. S1). The initial screening assays, followed by confirmatory measurements (Fig. 1A and B), revealed that methionine (Met) supplementation increased current generation 1.5-fold. To investigate the dose-dependent effect of Met supplementation, the current generation was measured in the presence of 0.013 and 1.3 µM Met (Fig. S2). Current generation increased significantly at 1.3 µM Met, showing a positive correlation between increasing Met concentrations and current generation. However, supplementation with 130 µM Met did not affect the growth of MR-1 in LMM under either aerobic or anaerobic fumarate-reducing conditions (Fig. S3). These observations suggest that Met, or its intracellular metabolite, acts as a signaling molecule that modulates the EET activity of MR-1, rather than serving merely as a growth-promoting factor in minimal media. Moreover, the addition of homocysteine (hCys), a direct precursor of Met that acts as a co-effector for MetR to activate the Met biosynthesis pathway (13, 14), negatively affected current generation by MR-1 (Fig. S1; Fig. 1A and B). This finding further supports the hypothesis that Met metabolism is linked to the regulation of EET in MR-1 cells.

### Effects of Met supplementation on the transcriptomic profile of MR-1

The above results suggest that Met supplementation in MR-1 induces a broad transcriptomic response, affecting the expression of a wider range of genes than the canonical Met-responsive regulon described in enteric bacteria (13, 14). To screen for Met-responsive genes in MR-1, we conducted a comparative transcriptomic analysis of MR-1 cells cultured in LMM with or without 130 µM Met. Total RNA was extracted from aerobically cultured cells to obtain a core data set of Met-responsive genes, minimizing the potential confounding effects associated with changes in anaerobic electron transfer activity. Out of 4,214 genes in the MR-1 genome, 599 were upregulated and 555 were downregulated in the presence of 130 µM Met, with statistical significance at *P* < 0.05 and a log_2_ fold change (log_2_ FC) threshold of ≥1 or ≤–1 (Data S1 and S2). Genes involved in Met biosynthesis (*met* genes) were most significantly downregulated under Met-supplemented conditions (Table 1 and Data S2), likely due to a feedback regulatory mechanism for Met biosynthesis (15). Further analysis focused on genes related to catabolic pathways revealed consistent upregulation of genes encoding EET components (*cymA*, *omcA-mtrCAB*, and *fccA*) and DMSO reductase (*dms* genes) in response to Met supplementation (Table 1, Data S1). The upregulation of *cymA* is particularly noteworthy, as CymA, the inner membrane-anchored periplasmic cytochrome *c* essential for EET, functions as a critical electron transfer hub in many anaerobic respiratory pathways, including those for fumarate, DMSO, and nitrate reduction (16). In contrast, TMAO reduction is independent of CymA (17), and the expression of genes specifically involved in TMAO reduction (*torECAD*) was not significantly altered by Met supplementation. In addition, Met supplementation upregulated *hem* and *ccm* genes, which are involved in heme biosynthesis (18) and cytochrome *c* maturation (19), respectively (Table 1, Data S1). Many genes encoding inner membrane-localized electron transfer proteins (e.g., *fdn*, *frd*, *nqr*, *lld*, and *dld*) and fermentative dehydrogenases (*ldhA* and *adhE*) were also upregulated (Table 1, Data S1). Collectively, these findings suggest the existence of a regulatory mechanism that coordinately activates the expression of genes involved in catabolic pathways, particularly those for anaerobic electron transfer, in response to Met availability.

**TABLE 1:** List of selected Met-responsive or MetR-regulated genes in MR-1

Process	Locus tag	Gene (operon)	Log_2_ FC^*a*^		TF^*c*^
			Met^+^/Met^−^	MR-1*metR*^+^/vc	
EET and anaerobic respiration	SO_0970	*fccA*	2.9	−2.3	nd
	SO_1427–1432	*dmsEFABGH*	3.3	−1.5	MetR, CRP
	SO_1779–1776	*omcA-mtrCAB*	2.4	−3.9	MetR, CRP
	SO_4591	*cymA*	1.3	−2.2	MetR, CRP
Heme biosynthesis	SO_0027	*hemG*	2.5	−3.1	nd
	SO_0435	*hemE*	1.2	−2.3	nd
	SO_1300	*hemL*	1.3	−1.8	nd
	SO_2019	*hemH*	2.8	−2.1	nd
	SO_3720	*hemG*	3.0	−1.3	nd
	SO_3834–3832	*hemA-prfA-prmC*	1.6	−1.1	MetR, CRP
	SO_4313–4316	*hemCDXY*	1.5	−1.4	nd
Cytochrome *c* maturation	SO_0263–0259	*ccmABCDE*	1.0	ns	nd
	SO_0265	*ccmI*	1.1	−2.3	MetR, CRP
	SO_0266–0269	*ccmFGH-*SO_0269	ns	−2.2	MetR, CRP
Intracellular electron transfer	SO_0101–0104	*fdnGHIE*	1.7	−2.4	CRP
	SO_0396–0399	*frdC1C2AB*	2.3	−3.6	CRP
	SO_0902–0907	*nqrABCDEF*	1.5	−3.8	nd
	SO_1520–1518	*lldEFG*	2.1	−1.4	MetR
	SO_1522–1521	SO_1522-*dld*	1.1	−3.0	CRP
	SO_3286–3285	*cydAB*	1.7	−4.1	CRP
Fermentation	SO_0968	*ldhA*	2.0	ns	CRP
	SO_2136	*adhE*	1.6	−1.3	CRP
Central carbon metabolism	SO_2490	*hexR*	1.0	−1.2	nd
	SO_2491	*pykA*	1.0	−2.3	CRP
	SO_2912–2913	*pflBA*	2.1	−3.9	nd
Ubiquinone biosynthesis	SO_2413	*ubiG*	1.2	ns^*b*^	nd
	SO_4199–4208	*ubiE–hemB*	1.2	−1.2	MetR
Methionine biosynthesis	SO_0817	*metR*	−4.4	6.5	MetR
	SO_0818	*metE*	−6.8	ns	MetR
	SO_1095	*metY*	−2.4	1.7	nd
	SO_1676	*metA*	−2.0	1.1	nd
	SO_4056	*metB*	−3.7	1.2	nd
Other amino acid metabolism	SO_1101	*luxS*	ns	2.6	MetR
	SO_0275–0279	*argCBFGH*	1.2	1.1	MetR
	SO_3019	*trpE*	−1.5	1.2	MetR
	SO_3471	*glyA*	−1.4	1.8	MetR

^*a*^ Log_2_-transformed fold change calculated from the ratio of gene expression levels in wild-type MR-1 cultured under Met-supplemented conditions to those under unsupplemented conditions (Met^+^/Met^−^) or from the ratio of gene expression levels in MR-1 metR^+^ to those in MR-1vc (MR-1metR^+^/vc). ns, not significantly changed. For the operons, the fold change of the first gene in each operon is used as the representative value.

^*b*^ Significantly changed in quantitative RT–PCR analysis (see Fig. 2).

^*c*^ Transcription factor (MetR or CRP) predicted based on motif analysis (Data S5 and S7). nd, not detected.

**Fig 2:**
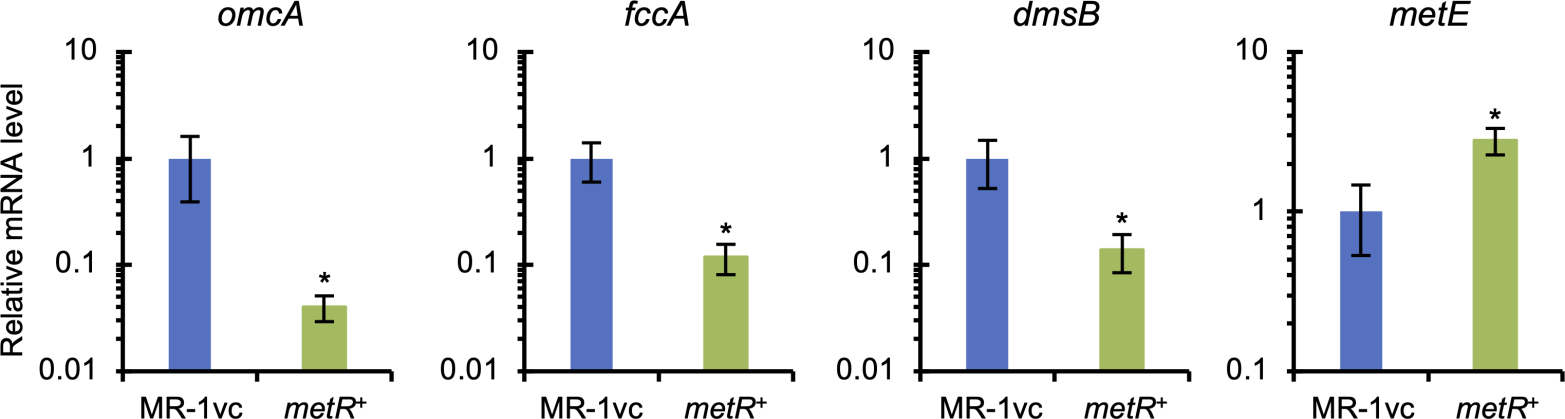
Differential expression of anaerobic respiration and methionine biosynthesis genes in MR-1*metR*^+^. The expression levels of *omcA*, *fccA*, *dmsB*, and *metE* were analyzed by quantitative RT-PCR using total RNA extracted from cells cultured anaerobically in LMM supplemented with TMAO as the electron acceptor. Results are presented as the relative levels of mRNA expression in MR-1vc cells. Bars and error bars represent means and standard deviations, respectively (*n* = 3 or 4 biological replicates). Asterisks indicate statistically significant differences (*P* < 0.05, Student’s *t*-test).

### Regulation of anaerobic electron transfer genes by MetR

**Fig 3:**
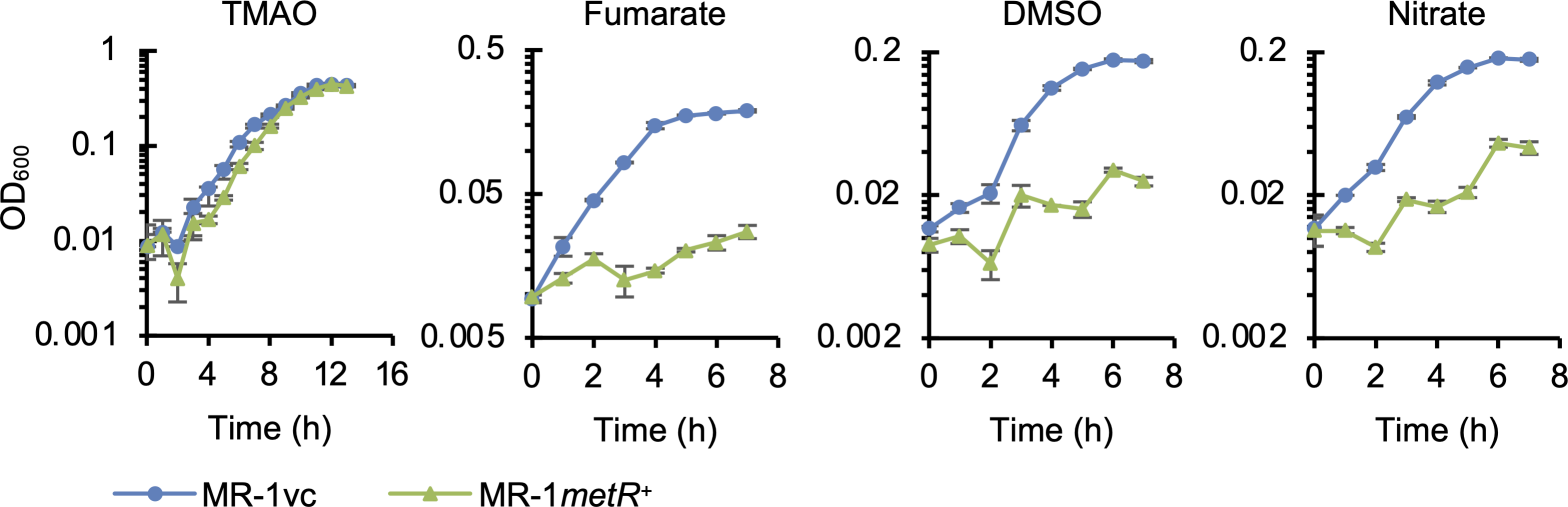
Growth of MR-1vc and MR-1*metR ^+^* under anaerobic respiration conditions. *Shewanella oneidensis* was cultured in LB medium supplemented with TMAO, fumarate, DMSO, or nitrate as the electron acceptor. Bars and error bars indicate means and standard deviations, respectively (*n* = 3 biological replicates).

The MR-1 genome encodes an ortholog of MetR, a transcriptional activator of *met* genes, which shares 46% amino acid identity with the well-characterized *Escherichia coli* MetR (20). We hypothesized that MetR negatively regulates the transcription of genes involved in EET and other anaerobic respiration pathways, given that current generation was enhanced by Met and suppressed by hCys, a co-effector of MetR (14) (Fig. 1A and B) and that Met supplementation significantly downregulated *metR* transcription (Table 1). To test this hypothesis, we constructed a *metR* deletion strain (∆*metR*), a complemented strain (Δ*metR*-C), and an overexpression strain (MR-1*metR*^+^) to characterize their physiological properties under anaerobic conditions. Growth assays under TMAO-reducing conditions in LMM (Fig. S4) and lysogeny broth (LB) medium (Fig. S5) revealed that the *metR* deletion strain (transformed with the control vector pBBR1MCS-5, Δ*metR*vc) exhibited a severe growth defect in the minimal medium, which was rescued in Δ*metR*-C. Given this growth defect of ∆*metR*, we focused subsequent analyses on characterizing MR-1*metR*^+^. Measurements in ECs (Fig. 1C and D) demonstrated that MR-1*metR*^+^ produced significantly lower current than the vector control strain (MR-1vc, harboring pBBR1MCS-5), although MR-1*metR*^+^ exhibited growth comparable to MR-1vc in LMM under TMAO-reducing conditions (Fig. S4). Additionally, we examined the growth of MR-1*metR*^+^ in the presence of different electron acceptors using LB (amino acid-rich) medium to minimize the influence of *metR* expression on specific metabolic pathways and associated growth phenotypes. MR-1*metR*^+^ exhibited markedly impaired growth under fumarate-, DMSO-, and nitrate-reducing conditions (Fig. 3) but comparable growth to MR-1vc under aerobic conditions (Fig. S6) and anaerobic TMAO-reducing conditions (Fig. 3). These findings indicate that MR-1*metR*^+^ has a diminished capacity for EET and fumarate, DMSO, and nitrate respiration.

To investigate the mechanism underlying the impaired current generation and anaerobic growth observed in MR-1*metR*^+^, we conducted a comparative transcriptomic analysis of MR-1*metR*^+^ and MR-1vc. Both strains were cultured anaerobically in LMM containing TMAO, an electron acceptor that MR-1*metR*^+^ can utilize (growth curves are shown in Fig. S4), to examine the *in vivo* regulatory function of MetR under anaerobic conditions. The overexpression of *metR* led to significant transcriptional changes in 722 genes, with 308 genes upregulated and 414 downregulated (Data S3 and S4). Notably, many differentially expressed genes in MR-1*metR*^+^ exhibited expression patterns opposite to those observed with Met supplementation. Specifically, many genes involved in EET and other catabolic pathways, as well as several *hem* and *ccm* genes, were downregulated in MR-1*metR*^+^, whereas *met* genes were largely upregulated (Table 1). This trend was further confirmed by quantitative RT-PCR analysis for four selected genes (*omcA*, *fccA*, *dmsB*, and *metE*) (Fig. 2). Taken together, these results suggest that the impaired current production and anaerobic growth observed in MR-1*metR*^+^ are caused by the transcriptional repression of genes involved in the EET and anaerobic respiratory pathways.

### Binding of MetR to the upstream regions of anaerobic electron transfer genes

**Fig 4:**
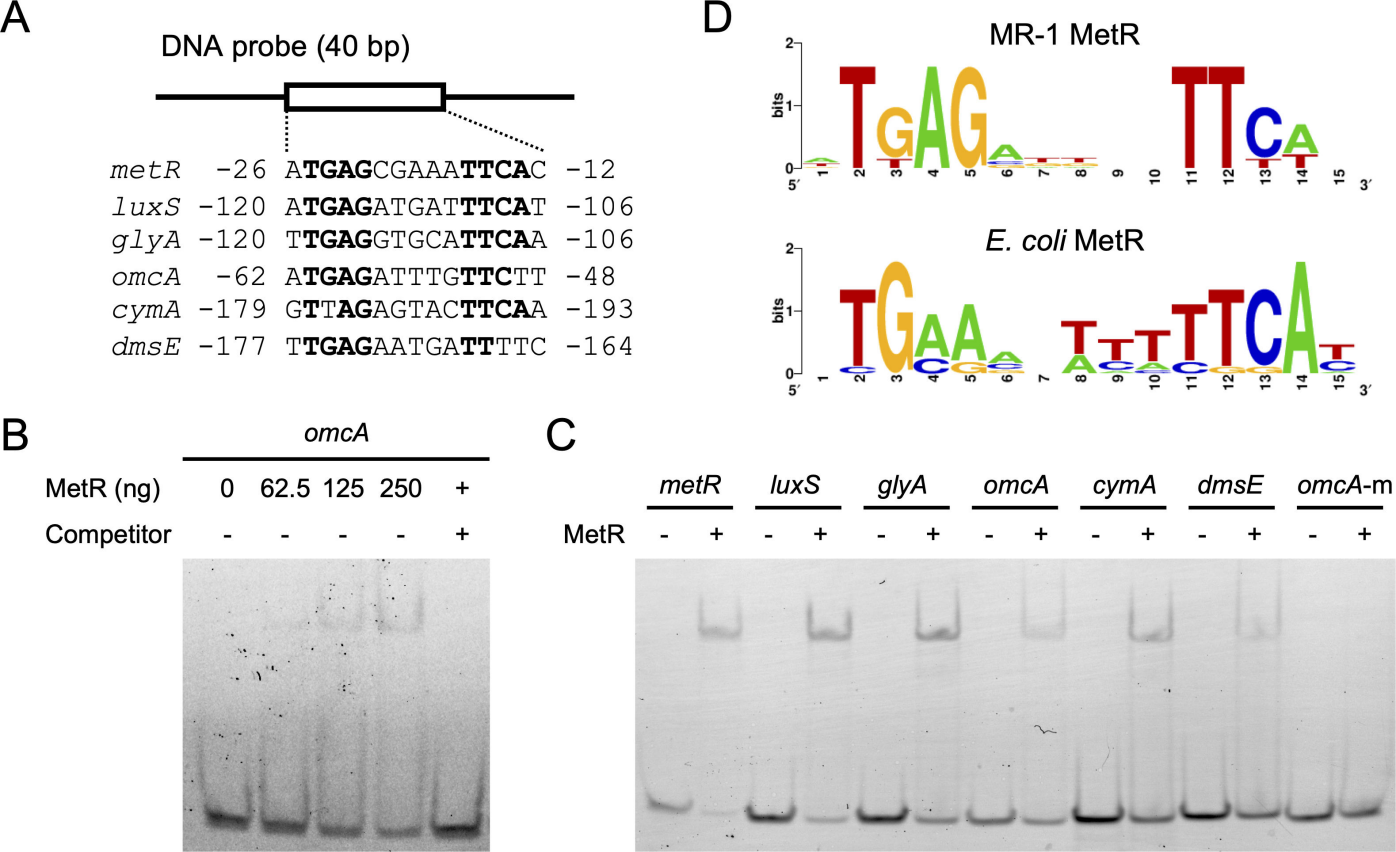
Binding of MetR to DNA regions upstream of the selected MetR-regulated genes. (**A**) DNA probes used in EMSA. Nucleotides commonly found in the MetR-binding sequences are shown in bold. Numbers on either side of each DNA sequence indicate positions relative to the start codon (+1) of the downstream gene. (**B**) EMSA using the MetR and *omcA* probes. The Cy-3-labeled probe was incubated with 0–250 ng of purified MetR in the presence (+) or absence (−) of a specific competitor (a 5,000-fold excess of unlabeled *omcA* probe). (**C**) EMSA using DNA probes of selected genes involved in anaerobic respiration and methionine metabolism. Labeled probes were incubated in the presence (+) or absence (−) of 500 ng of purified MetR. The mutated *omcA* probe (*omcA*-m), in which the palindromic eight nucleotides in the 13-bp MetR-binding consensus sequence were substituted with guanine residues, was used as a negative control. (**D**) Site-specific nucleotide frequencies in the MetR-binding DNA sequences. The sequence logo representing the MetR-binding sequences in MR-1 was generated using the six sequences shown in panel A. For comparison, the sequence logo for known MetR-binding sequences in *E. coli* K-12 is shown. The height of each letter represents the degree of sequence conservation measured in bits.

We performed electrophoretic mobility shift assays (EMSAs) to investigate the direct regulation of EET and anaerobic respiratory genes by MetR (Fig. 4). A 13-bp palindromic consensus sequence for MetR binding (5′-TGAANNNNNTTCA-3′) has been reported in *E. coli*, *Salmonella typhimurium*, and *Vibrio harveyi* (21, 22). Similar sequences were found in the upstream region of *metR* (i.e., the *metR–metE* intergenic region) and in the upstream regions of several differentially expressed genes in MR-1*metR*^+^, including *luxS*, *glyA*, *omcA*, *cymA*, and *dmsE*. Based on the upstream regions of these six genes, we generated 40-bp DNA probes containing the putative 13-bp MetR-binding sequences (Fig. 4A) and examined the binding of purified MetR (Fig. S7). When MetR was incubated with the *omcA* probe, shifted bands were observed in a protein concentration-dependent manner, whereas the shift disappeared in the presence of a specific DNA competitor (excess unlabeled *omcA* probe) (Fig. 4B). Similar shifts were observed when MetR was incubated with the other five DNA probes, whereas no shift was detected with a mutated *omcA* probe, in which the palindromic nucleotides had been replaced (Fig. 4C). These results demonstrate that MetR directly binds to the upstream regions of the tested genes containing the predicted MetR-binding sequences.

### Prediction of the MetR regulon in MR-1

Based on the six MetR-binding sequences identified by EMSA (Fig. 4A), we constructed a position-specific scoring matrix (PSSM) for the consensus MetR-binding sequence in MR-1 and visualized it as a sequence logo (Fig. 4D). The 13-bp MR-1 MetR-binding sequence (consensus: 5′-TGAGNNNNNTTCA-3′) was largely identical to that of *E. coli*, although the substitution of the fourth nucleotide from A to G slightly disrupted palindromic symmetry. Using the PSSM, we scanned the MR-1 genome (500-bp upstream regions of each gene) and identified putative MetR-binding sites for 151 genes (Data S5). Of these, 37 genes were differentially expressed in MR-1*metR*^+^ (19 upregulated and 18 downregulated). Based on this information and co-transcription patterns (Data S3 and S4), we predicted the MetR regulon in the MR-1 genome (Data S6). Notably, this regulon includes several *hem* and *ccm* genes, as well as *cymA*, *omcA-mtrCAB*, and *dmsEFABGH*, which were consistently downregulated in MR-1*metR*^+^. The genes involved in TMAO reduction (*torECAD*) (23, 24) are not part of this regulon, consistent with the ability of MR-1*metR*^+^ to grow under TMAO-reducing conditions (Fig. 3). The analysis also predicted that MetR directly activates the transcription of genes for the metabolism of arginine (*argCBFGH*), phenylalanine (*pheA*), tryptophan (*trpE*), glycine (*glyA*), and methionine (*metE* and *luxS*). These results suggest that MetR functions as a global regulator, repressing anaerobic electron transfer genes while activating various amino acid metabolism genes.

MR-1 uses CRP to activate the transcription of genes for EET and other anaerobic respiratory systems, excluding TMAO reduction (9). To explore the overlap between the MetR and CRP regulons, we scanned the MR-1 genome using a PSSM for the CRP-binding sequences identified in *E. coli*, given that MR-1 CRP shares 89% amino acid identity with *E. coli* CRP. This analysis identified putative CRP-binding sites in the upstream regions of 369 genes (Data S7), 12 of which, including *omcA*, *dmsE*, *cymA*, and *ccmF*, overlapped with the MetR regulon (Table 1, Data S6). The experimentally confirmed CRP-binding site upstream of *omcA* (10) is located upstream of the MetR-binding site (Fig. S8). Similar motif arrangements are present upstream of *cymA*, *dmsE*, and *ccmF* (Data S6). These arrangements, together with the observed downregulation of these genes in MR-1*metR*^+^ (Table 1; Figure 2), suggest that MetR represses their transcription by interfering with CRP-dependent activation.

### Inhibition of EET by *metR* expression in *Aeromonas hydrophila*

**Fig 5:**
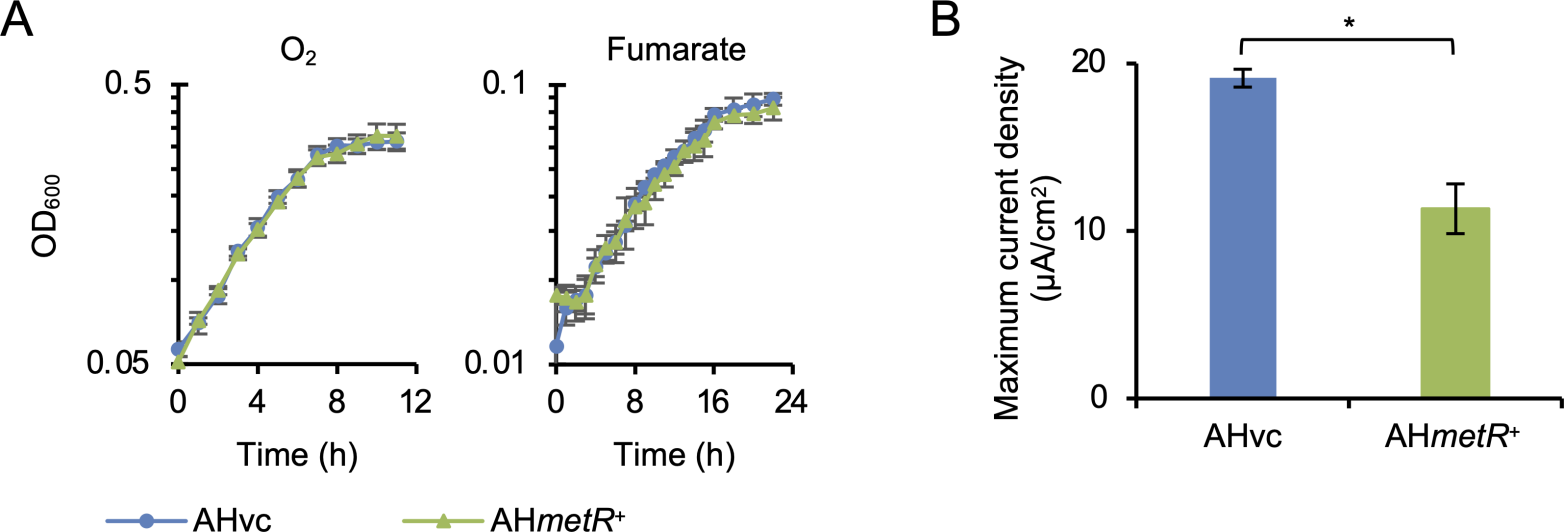
Effect of *metR* expression on the respiratory activity *of Aeromonas hydrophila*. (**A**) Growth of AHvc and AH*metR*^+^ in LMM under aerobic (O_2_-reducing) and fumarate-reducing conditions. (**B**) Maximum current densities in ECs inoculated with AHvc and AH*metR*^+^. ECs were operated at a working electrode potential of +0.4 V (vs the standard hydrogen electrode). In all panels, error bars represent standard deviations (*n* = 3 biological replicates). The asterisk indicates statistically significant differences (*P* < 0.05, Student’s *t*-test).

Given that MetR is a transcription factor widely conserved across proteobacteria (15), we hypothesized that MetR-dependent repression of anaerobic electron transfer is conserved in closely related bacteria. To test this hypothesis, we engineered *Aeromonas hydrophila* ATCC 7966, a bacterium with EET activity (25) and relatively close phylogenetic proximity to *Shewanella* (26), to overexpress its native *metR* (AHA_1955). Elevated *metR* expression in the resulting strain (AH*metR*^+^) was confirmed by quantitative RT-PCR analysis (Fig. S9A). When cultured in LMM under aerobic and fumarate-reducing conditions, AH*metR*^+^ exhibited comparable growth to the vector control strain (AHvc) (Fig. 5A). However, AH*metR*^+^ produced lower current in ECs compared to AHvc (Fig. 5B), demonstrating that *metR* expression negatively affects EET in *A. hydrophila*. For comparison, we constructed an *E. coli* strain overexpressing its native *metR* (EC*metR*^+^) (Fig. S9B). EC*metR*^+^ exhibited comparable growth to the vector control strain (ECvc) under fumarate- and nitrate-reducing conditions (Fig. S10), suggesting that MetR does not regulate these anaerobic respiratory pathways in *E. coli*.

Scanning of the *A. hydrophila* genome using the PSSM based on MR-1 MetR-binding sequences identified a putative binding sequence upstream of *ccmF but* not in the upstream regions of the known EET genes *mtrCAB* (AHA_2764–2766), *pdsA* (AHA_2763), and *netBCD* (AHA_2762-2760) (25) (Data S8). These results, together with the comparatively weaker repression of EET in *A. hydrophila* relative to that observed in MR-1 (Fig. 1 and 5B), suggest that the influence of MetR on the repression of anaerobic electron transfer in *A. hydrophila* is more limited than its effects in MR-1.

## DISCUSSION

In *E. coli* and *Salmonella*, MetR activates the transcription of the methionine synthase gene *metE* in the presence of hCys as a co-effector (14, 21). In these enteric bacteria, *metR* and *metE* are located adjacently in opposite orientations, with overlapping promoter regions. Consequently, MetR activates *metE* transcription while repressing its own expression. In addition to this autoregulatory mechanism, *metR* transcription is repressed by MetJ and its co-repressor, *S*-adenosylmethionine (SAM) (13). Synthesized from methionine, SAM enhances the DNA-binding activity of MetJ, thereby repressing the transcription of MetJ-regulated genes, including *metR* and other *met* genes (e.g., *metA*, *metB*, *metC*, *metE*, and *metF*) (27). These regulatory systems ensure that *met* expression is upregulated in response to low intracellular Met levels, specifically when its direct precursor, hCys, is available. In MR-1, *metR* and *metE* are similarly arranged, and the intergenic region contains a MetR-binding sequence (Fig. 4). The deletion of *metR* in this strain led to growth inhibition in minimal medium (Fig. S4), consistent with observations in *E. coli* (28). Together with the transcriptomic profiles shown in Table 1, these findings suggest that MR-1 employs regulatory systems for *met* genes analogous to those conserved in enteric bacteria.

**Fig 6:**
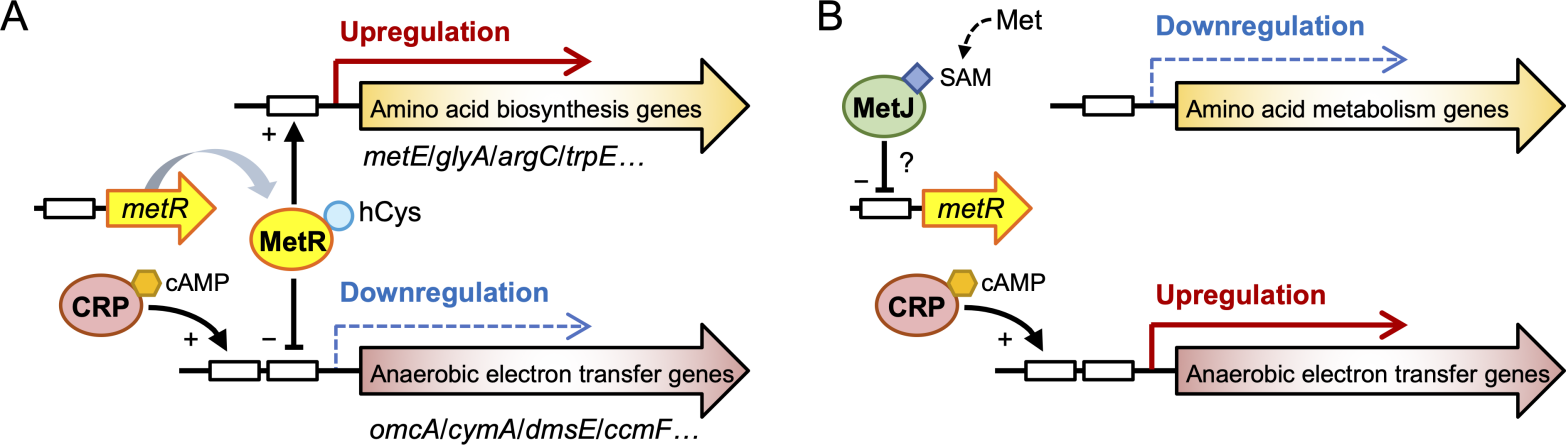
Proposed mechanisms of MetR-mediated regulation of anaerobic electron transfer and amino acid biosynthesis pathways. (**A**) Regulation under Met-limited conditions. (**B**) Regulation under Met-rich conditions. Boxes represent upstream regulatory regions bound by transcription factors. +, positive regulation; −, negative regulation.

We propose that MetR functions as a molecular switch balancing the expression of amino acid biosynthesis and anaerobic respiratory pathways (Fig. 6). Under Met-limited conditions, the transcriptional repression of *metR* is relieved, allowing MetR to activate the transcription of *metE* and several other amino acid biosynthesis genes while simultaneously repressing genes involved in anaerobic electron transfer and the synthesis of heme and *c*-type cytochromes (Fig. 6A). Given that hCys inhibited the EET activity of MR-1 (Fig. 1A and B), hCys likely acts as a co-repressor in this MetR-dependent repression. Conversely, under Met-rich conditions, *metR* expression is downregulated, leading to the transcriptional activation (derepression) of anaerobic electron transfer genes (Fig. 6B). Although Met supplementation upregulated genes involved in fumarate reduction, such as *cymA* and *fccA*, it did not enhance cell growth under fumarate-reducing conditions (Fig. S3). This is likely because growth on fumarate was limited by anabolic processes or cell division rather than the respiratory electron transfer rate.

The MetR-mediated regulatory system for anaerobic electron transfer genes may represent a survival strategy that bacteria have evolved to optimize energy allocation in response to nutrient availability. Met is the most energetically expensive amino acid to synthesize under anaerobic conditions (29). Additionally, anaerobic respiratory bacteria with EET pathways must expend considerable energy to synthesize multi-heme cytochromes (30, 31). Thus, when exogenous Met is available, it is plausible that these bacteria repress Met biosynthesis and allocate energy to heme and cytochrome *c* synthesis to maximize anaerobic respiration. Conversely, when Met is scarce, they likely prioritize Met biosynthesis while downregulating anaerobic electron transfer.

The MR-1 MetR regulon (Data S6) is notably distinct from that of other proteobacteria (15). It includes not only *met*, *luxS*, and *glyA* but also *arg* and *trpE*, as well as key anaerobic respiratory genes, supporting the idea that this strain employs MetR as a global regulator for amino acid biosynthesis and anaerobic respiration. A subset of this regulon, including *cymA*, *omcA*, *dmsE*, *ccmI*, and *ccmF*, overlaps with the CRP regulon (Table 1; Data S6). Based on our data and current knowledge of CRP function (9, 10), these genes are likely upregulated by CRP and downregulated by MetR. A similar competitive regulatory mechanism exists in *V. harveyi* (22), where CRP and MetR, together with the quorum-sensing regulator LuxR, bind to the *lux* promoter, thereby modulating bioluminescence in response to nutrient availability and cell density. Similarly, MR-1 may use CRP and MetR to enable flexible and fine-tuned control of respiratory activity in response to nutrient and energy fluctuations.

Our transcriptomic analysis revealed that Met supplementation or *metR* overexpression affects the expression of various genes involved in inner membrane electron transfer, lactate and ethanol fermentation, and central carbon metabolism (Table 1). These findings suggest that elevated intracellular Met levels trigger the coordinated activation of genes involved in intracellular catabolic processes, as well as EET and specific anaerobic pathways. Notably, motif-based predictions suggest that many of these Met-responsive catabolic genes are directly regulated by CRP, rather than MetR (Table 1 and Data S7). Although the underlying molecular mechanisms remain unclear, it is possible that an unknown transcription factor, acting downstream of Met signaling, regulates these genes in coordination with CRP. Many of these putative CRP-regulated genes, including those for formate dehydrogenase (*fdn*), quinol:fumarate reductase (*frd*), respiratory D-lactate dehydrogenase (*dld*), and ubiquinol oxidase (*cyd*), have been reported to be upregulated under oxygen-limited conditions (32). Furthermore, the expression of genes encoding the Na^+^-dependent NADH dehydrogenase complex (*nqr*) and ubiquinone biosynthesis proteins (*ubiG* and *ubiE*), which are predicted to be CRP independent, is also known to be induced under oxygen-limited conditions (32). Although our transcriptomic analysis with Met supplementation was conducted under aerobic conditions, the observed transcriptomic responses suggest that elevated intracellular Met levels promote a metabolic shift from aerobic to anaerobic metabolism in MR-1. These results support the idea that intracellular Met levels serve as a critical signal for the global and coordinated regulation of catabolic processes, particularly carbon catabolism and electron transfer under anaerobic conditions.

MetR is involved in diverse physiological processes in proteobacteria, including virulence regulation in *Vibrio cholerae* (33), secondary metabolite synthesis in *Serratia marcescens* (34), and swarming motility in *Pseudomonas aeruginosa* (35). Our data suggest that MetR also regulates EET in *A. hydrophila* (Fig. 5B). However, predictions of MetR-binding sequences suggest that in this bacterium, MetR regulates *ccm* genes rather than genes encoding structural EET components (Data S8). Given that EET requires abundant cytochrome *c* proteins (30, 31), impaired cytochrome *c* maturation could explain the decreased current production in AH*metR*^+^, despite the absence of growth defects under either aerobic or anaerobic fumarate-reducing conditions (Fig. 5A). No inhibition of anaerobic growth was observed in EC*metR*^+^, consistent with the absence of genes for anaerobic respiration or cytochrome *c* synthesis in the *E. coli* MetR regulon (15). Further research is needed to determine if the regulation of anaerobic electron transfer by MetR is conserved in a taxonomic group that includes *Shewanella* and *Aeromonas*, such as the VAAP subgroup of Gammaproteobacteria (26), or if this regulation is specific to bacteria that require fine-tuned cytochrome *c* synthesis for EET.

In conclusion, our results suggest that Met serves not only as an essential building block for protein synthesis but also as a signal that regulates bacterial catabolic activity. This finding is relevant to optimizing METs using *Shewanella* and related species, as the composition and concentrations of amino acids in the medium can affect the performance of bioelectrochemical reactors by modulating bacterial EET activity. However, our results do not exclude the possibility that regulatory factors other than CRP and MetR contribute to the expression of EET-related genes in MR-1. Further studies are needed to elucidate the complex regulatory network governing the catabolic activity of this model bacterium.

## MATERIALS AND METHODS

### Bacterial strains and culture conditions

Strains and plasmids are listed in Table S1. *S. oneidensis* and *A. hydrophila* were cultured at 30°C in LB medium or minimal medium (11) containing 10 mM lactate (LMM). For *A. hydrophila*, LMM was supplemented with 0.1 g/L yeast extract. *E. coli* was cultured at 37°C in LB medium or M9 minimal medium (36) containing 20 mM glycerol and 0.5 mM isopropyl β-D-1-thiogalactopyranoside (IPTG) to induce *lac* promoter expression. Glycerol was used as a substrate to inhibit fermentative growth in the absence of external electron acceptors. For aerobic cultures, 5 mL of LB, LMM, or M9 glycerol medium in a test tube (30 mL capacity) was inoculated with a bacterial strain at an initial optical density at 600 nm (OD_600_) of 0.05 and was shaken at 180 rpm. For anaerobic cultures, 5 or 80 mL of medium in a screw-top test tube (10 mL capacity, for growth curve analysis) or vial (100 mL capacity for transcriptome analysis), respectively, was supplemented with 20 mM TMAO, fumarate, DMSO, or nitrate as the electron acceptor and inoculated with a bacterial strain at an initial OD_600_ of 0.01. A test tube or vial containing the inoculated medium was sealed with a butyl rubber septum, purged with high-purity nitrogen (99.99%), and incubated without shaking. When necessary, 50 µg/mL kanamycin (Km) or 15 μg/mL gentamicin was added to the culture medium.

### Operation of EC

A single-chamber three-electrode EC with a capacity of 18 mL (12) was used to monitor the electric current generated by *S. oneidensis* and *A. hydrophila* strains. The EC was equipped with a graphite felt working electrode (2.25 cm^2^), an Ag/AgCl reference electrode (HX-R5; Hokuto Denko, Tokyo, Japan), and a titanium mesh counter electrode (4 cm^2^, φ0.1 mm, 100 mesh/inch; Nilaco, Tokyo, Japan). The EC was filled with 15 mL of LMM supplemented with 170 mM NaCl as electrolyte, inoculated with bacterial cells at an initial OD_600_ of 0.01, and purged with high-purity nitrogen (99.99%). The electrodes were connected to a potentiostat (VMP3; Biologic, Claix, France), and the working electrode was poised at +0.2 V vs Ag/AgCl (equivalent to +0.4 V vs the standard hydrogen electrode). The system was operated at 30°C under ambient atmospheric conditions. Current density (A/cm^2^) was calculated based on the projected area of the working electrode.

### Mutant construction

In-frame disruption of *metR* (SO_0817) in MR-1 was performed using a two-step homologous recombination method with the suicide plasmid pSMV10 (37, 38). In brief, a 1.6-kb fusion product, consisting of upstream and downstream sequences of *metR* joined by an 18-bp linker sequence, was constructed by PCR and *in vitro* extension using total DNA from MR-1 and primers (listed in Table S2). The amplified fusion product was ligated into the SpeI site of pSMV10. The resulting plasmid, pSMV-metR, was introduced into MR-1 by filter mating with *E. coli* WM6026, and transconjugants were screened as previously described (37, 38), to isolate double-crossover mutants. The disruption of the target gene in the resulting strains was confirmed by PCR. A representative mutant strain in which *metR* was deleted was selected and designated ∆*metR*.

To construct complementation and overexpression plasmids, *metR* fragments were PCR-amplified from the host genomes using the primers listed in Table S2. The MR-1 *metR* fragment was digested with EcoRI and SpeI and cloned into the corresponding sites of pBBR1MCS-5 (39). The resulting plasmid, pBBR-SOmetR, was introduced into wild-type MR-1 and ∆*metR* by filter mating, and the transformants were designated MR-1*metR*^+^ and ∆*metR*-C, respectively. Similarly, *metR* fragments from *A. hydrophila* ATCC 7966 and *E. coli* DH5α were digested with EcoRI and BamHI (for *A. hydrophila*) or EcoRI and PstI (for *E. coli*) and cloned into the corresponding sites of pBBR1MCS-5 to generate pBBR-AHmetR and pBBR-ECmetR, respectively. The insert sequences of all constructed plasmids were verified by DNA sequencing. These plasmids were introduced into *A. hydrophila* or *E. coli* DH5α to generate AH*metR*^+^ and EC*metR*^+^, respectively. Strains transformed with the empty vector pBBR1MCS-5 were designated AHvc and ECvc, respectively.

### RNA extraction

For the transcriptomic profiling of MR-1 under Met-supplemented and Met-unsupplemented conditions, cells (*n* = 3 biological replicates) were cultured aerobically in LMM supplemented with or without 130 µM Met and harvested at the logarithmic growth phase (OD_600_ of 0.2–0.3). For MR-1*metR*^+^ and MR-1vc profiling, cells (*n* = 4) were cultured anaerobically in LMM containing 20 mM TMAO as the electron acceptor and harvested at the logarithmic growth phase (OD_600_ of 0.13–0.15). *A. hydrophila* strains (AH*metR*^+^ and AHvc) were cultured aerobically in LMM (*n* = 3) and harvested at the logarithmic growth phase (OD_600_ of 0.15–0.25). *E. coli* strains (EC*metR*^+^ and ECvc) were cultured anaerobically in M9 glycerol medium supplemented with 20 mM fumarate and 0.5 mM IPTG (*n* = 3) and harvested at the logarithmic growth phase (OD_600_ of 0.05–0.07). Total RNA was extracted using Trizol reagent (Thermo Fisher Scientific), purified using RNeasy Mini Kit and RNase-free DNase Set (Qiagen, Valencia, CA, USA), and evaluated for purity using Agilent 2100 Bioanalyzer with RNA 6000 Pico reagents and RNA Pico Chips (Agilent Technologies, Santa Clara, CA, USA), according to the manufacturer’s instructions.

### Transcriptome analyses

Transcriptome analysis was performed using a custom DNA microarray for MR-1 (8 × 15K, Agilent Technologies), which was designed (40) and validated (12, 41–43) in other studies. Cyanine 3 (Cy3)-labeled complementary RNA was synthesized from 50 ng of total RNA using the Low Input Quick Amp WT Labeling Kit (Agilent Technologies). Gene expression data (*n* = 3–4 biological replicates) were normalized and statistically analyzed using the limma software package (version 3.36.2) for R (44). A paired Student *t*-test followed by the Benjamini–Hochberg false discovery rate correction was used for statistical analyses. Differential expression for each probe was considered statistically significant when the absolute value of log_2_ FC was >1.0 at *P* < 0.05.

### Quantitative RT-PCR

Quantitative RT-PCR was performed using a LightCycler 1.5 instrument (Roche, Indianapolis, IN, USA), as previously described (45). Briefly, the PCR reaction mixture contained 15 ng of total RNA, 1.3 µL of 50 mM Mn(OAc)_2_ solution, 7.5 µL of LightCycler RNA Master SYBR Green I (Roche), and 0.15 µM primers (listed in Table S2). The expression levels of the target genes were normalized to the expression level of the 16S rRNA gene.

### Purification of MetR

To construct a plasmid expressing MetR with a histidine tag at the C-terminus (C-his-MetR), the *metR* gene was PCR-amplified from the total DNA of MR-1 using the primers listed in Table S2. The PCR product was digested with NdeI and XhoI and cloned between the corresponding sites of the pET-26b(+) vector (Merck, Darmstadt, Germany). The resulting plasmid, pET-metR (Table S1), was introduced into *E. coli* BL21(DE3). The transformed cells were cultured at 30°C in 100 mL 2× yeast extract-tryptone medium supplemented with Km. IPTG was added to a final concentration of 0.5 mM when the OD_600_ reached 0.3. After overnight culture at 16°C, the cells were harvested by centrifugation. C-his-MetR was then extracted by ultrasonication and purified using QuickPick IMAC Metal Affinity Kit for Proteins (Bio-Nobile, Turku, Finland), as previously described (46).

### EMSA

EMSA was performed as previously described (47), with some modifications. Cy3-labeled 40-bp DNA probes were generated by annealing complementary single-strand oligonucleotides (listed in Table S2). DNA-binding reactions were performed in a 20 μL reaction mixture containing 50 mM Tris-HCl (pH 8.0), 0.5 mM EDTA (pH 8.0), 2 mM MgSO_4_, 1 mM dithiothreitol, 100 µg/mL bovine serum albumin, 10 μg/mL poly(dI-dC) (Sigma-Aldrich), 4 mM hCys, 20% (vol/vol) glycerol, 1 nM Cy3-labeled DNA probe, and 0–500 ng of C-his-MetR. The mixture was incubated at 20°C for 10 min and loaded onto a nondenaturing 5%–20% gradient polyacrylamide gel (E-T520L; ATTO, Tokyo, Japan). Electrophoresis was performed at 200 V at 4°C in Tris-borate-EDTA buffer for 40 min. Fluorescence gel images were obtained using Typhoon FLA 9000 (GE Healthcare, Milwaukee, WI, USA).

### *In silico* prediction of regulatory sequences

The PSSM for MetR-binding sequences in MR-1 was constructed from the 13-bp motifs identified by EMSA using the PSSM-convert program (48). The PSSM for the CRP-binding sequences was obtained from the RegulonDB database (49). To identify putative MetR- or CRP-binding sites, 500-bp upstream sequences of the individual genes in MR-1 or *A. hydrophila* were scanned with each PSSM using the matrix-scan function of Regulatory Sequence Analysis Tools (50) with default parameters. Sequence logos were generated to visualize the PSSMs using WebLogo (51). The *E. coli* MetR-binding motif was generated using 11 known MetR-binding sequences in *E. coli* K-12, which were obtained from the RegPrecise database (52).

### Statistical analysis

Data were statistically evaluated using Student’s *t*-test or one-way analysis of variance followed by Tukey’s honestly significant difference test using JMP Pro 14.1.0 software (SAS Institute, Cary, NC, USA). Differences were considered statistically significant at a *P* value of <0.05.

## Data Availability

The authors declare that all data supporting the findings of this study are available within the article and its supplementary information files or from the corresponding author upon reasonable request. The microarray data obtained in this study have been deposited in the NCBI Gene Expression Omnibus under accession numbers GSE282263 and GSE282264.

## SUPPLEMENTAL MATERIAL

Data S1 jb.00181-26-s0001.xlsx Upregulated genes in MR-1 cells grown under Met-supplemented conditions.

Data S2 jb.00181-26-s0002.xlsx Downregulated genes in MR-1 cells grown under Met-supplemented conditions.

Data S3 jb.00181-26-s0003.xlsx Upregulated genes in MR-1 metR⁺.

Data S4 jb.00181-26-s0004.xlsx Downregulated genes in MR-1 metR+.

Data S5 jb.00181-26-s0005.xlsx Putative MetR-binding sites in the MR-1 genome.

Data S6 jb.00181-26-s0006.xlsx Predicted MetR regulon in MR-1.

Data S7 jb.00181-26-s0007.xlsx Putative CRP-binding sites in the MR-1 genome.

Data S8 jb.00181-26-s0008.xlsx Putative MetR-binding sites in the *A. hydrophila* genome.

Supplemental figures and tables jb.00181-26-s0009.pdf Figures S1 to S10, and Tables S1 and S2.

ASM does not own the copyrights to Supplemental Material that may be linked to, or accessed through, an article. The authors have granted ASM a non-exclusive, world-wide license to publish the Supplemental Material files. Please contact the corresponding author directly for reuse.

## References

[1] Russell JB, Cook GM. 1995. Energetics of bacterial growth: balance of anabolic and catabolic reactions. Microbiol Rev 59:48–62. doi:10.1128/mr.59.1.48-62.1995 7708012 PMC239354

[2] Decker K, Jungermann K, Thauer RK. 1970. Energy production in anaerobic organisms. Angew Chem Int Ed Engl 9:138–158. doi:10.1002/anie.197001381 4984685

[3] Chubukov V, Gerosa L, Kochanowski K, Sauer U. 2014. Coordination of microbial metabolism. Nat Rev Microbiol 12:327–340. doi:10.1038/nrmicro3238 24658329

[4] Venkateswaran K, Moser DP, Dollhopf ME, Lies DP, Saffarini DA, MacGregor BJ, Ringelberg DB, White DC, Nishijima M, Sano H, Burghardt J, Stackebrandt E, Nealson KH. 1999. Polyphasic taxonomy of the genus Shewanella and description of Shewanella oneidensis sp. nov. Int J Syst Evol Microbiol 49:705–724. doi:10.1099/00207713-49-2-705 10319494

[5] Nealson KH, Saffarini D. 1994. Iron and manganese in anaerobic respiration: environmental significance, physiology, and regulation. Annu Rev Microbiol 48:311–343. doi:10.1146/annurev.mi.48.100194.001523 7826009

[6] Fredrickson JK, Romine MF, Beliaev AS, Auchtung JM, Driscoll ME, Gardner TS, Nealson KH, Osterman AL, Pinchuk G, Reed JL, Rodionov DA, Rodrigues JLM, Saffarini DA, Serres MH, Spormann AM, Zhulin IB, Tiedje JM. 2008. Towards environmental systems biology of Shewanella. Nat Rev Microbiol 6:592–603. doi:10.1038/nrmicro1947 18604222

[7] Kouzuma A. 2021. Molecular mechanisms regulating the catabolic and electrochemical activities of Shewanella oneidensis MR-1. Biosci Biotechnol Biochem 85:1572–1581. doi:10.1093/bbb/zbab088 33998649

[8] Ikeda S, Takamatsu Y, Tsuchiya M, Suga K, Tanaka Y, Kouzuma A, Watanabe K. 2021. Shewanella oneidensis MR-1 as a bacterial platform for electro-biotechnology. Essays Biochem 65:355–364. doi:10.1042/EBC20200178 33769488 PMC8314016

[9] Saffarini DA, Schultz R, Beliaev A. 2003. Involvement of cyclic AMP (cAMP) and cAMP receptor protein in anaerobic respiration of Shewanella oneidensis. J Bacteriol 185:3668–3671. doi:10.1128/JB.185.12.3668-3671.2003 12775705 PMC156221

[10] Kasai T, Kouzuma A, Nojiri H, Watanabe K. 2015. Transcriptional mechanisms for differential expression of outer membrane cytochrome genes omcA and mtrC in Shewanella oneidensis MR-1. BMC Microbiol 15:68. doi:10.1186/s12866-015-0406-8 25886963 PMC4417206

[11] Ikeda S, Tomita K, Nakagawa G, Kouzuma A, Watanabe K. 2023. Supplementation with amino acid sources facilitates fermentative growth of Shewanella oneidensis MR-1 in defined media. Appl Environ Microbiol 89:e0086823. doi:10.1128/aem.00868-23 37367298 PMC10370299

[12] Hirose A, Kasai T, Aoki M, Umemura T, Watanabe K, Kouzuma A. 2018. Electrochemically active bacteria sense electrode potentials for regulating catabolic pathways. Nat Commun 9:1083. doi:10.1038/s41467-018-03416-4 29540717 PMC5852097

[13] Hondorp ER, Matthews RG. 2006. Methionine. EcoSal Plus 2:10. doi:10.1128/ecosalplus.3.6.1.7 26443567

[14] Urbanowski ML, Stauffer GV. 1989. Role of homocysteine in metR-mediated activation of the metE and metH genes in Salmonella typhimurium and Escherichia coli. J Bacteriol 171:3277–3281. doi:10.1128/jb.171.6.3277-3281.1989 2656646 PMC210046

[15] Leyn SA, Suvorova IA, Kholina TD, Sherstneva SS, Novichkov PS, Gelfand MS, Rodionov DA. 2014. Comparative genomics of transcriptional regulation of methionine metabolism in Proteobacteria. PLoS ONE 9:e113714. doi:10.1371/journal.pone.0113714 25411846 PMC4239095

[16] Cordova CD, Schicklberger MFR, Yu Y, Spormann AM. 2011. Partial functional replacement of CymA by SirCD in Shewanella oneidensis MR-1. J Bacteriol 193:2312–2321. doi:10.1128/JB.01355-10 21378180 PMC3133100

[17] Myers CR, Myers JM. 1997. Cloning and sequence of cymA, a gene encoding a tetraheme cytochrome c required for reduction of iron(III), fumarate, and nitrate by Shewanella putrefaciens MR-1. J Bacteriol 179:1143–1152. doi:10.1128/jb.179.4.1143-1152.1997 9023196 PMC178810

[18] Dailey HA, Dailey TA, Gerdes S, Jahn D, Jahn M, O’Brian MR, Warren MJ. 2017. Prokaryotic heme biosynthesis: multiple pathways to a common essential product. Microbiol Mol Biol Rev 81:e00048-16. doi:10.1128/MMBR.00048-16 28123057 PMC5312243

[19] Jin M, Jiang Y, Sun L, Yin J, Fu H, Wu G, Gao H. 2013. Unique organizational and functional features of the cytochrome c maturation system in Shewanella oneidensis. PLoS One 8:e75610. doi:10.1371/journal.pone.0075610 24040415 PMC3769277

[20] Weissbach H, Brot N. 1991. Regulation of methionine synthesis in Escherichia coli. Mol Microbiol 5:1593–1597. doi:10.1111/j.1365-2958.1991.tb01905.x 1943695

[21] Urbanowski ML, Stauffer GV. 1989. Genetic and biochemical analysis of the MetR activator-binding site in the metE metR control region of Salmonella typhimurium. J Bacteriol 171:5620–5629. doi:10.1128/jb.171.10.5620-5629.1989 2676984 PMC210406

[22] Chatterjee J, Miyamoto CM, Zouzoulas A, Lang BF, Skouris N, Meighen EA. 2002. MetR and CRP bind to the Vibrio harveyi lux promoters and regulate luminescence. Mol Microbiol 46:101–111. doi:10.1046/j.1365-2958.2002.03128.x 12366834

[23] Dos Santos JP, Iobbi-Nivol C, Couillault C, Giordano G, Méjean V. 1998. Molecular analysis of the trimethylamine N-oxide (TMAO) reductase respiratory system from a Shewanella species. J Mol Biol 284:421–433. doi:10.1006/jmbi.1998.2155 9813127

[24] Lemaire ON, Honoré FA, Jourlin-Castelli C, Méjean V, Fons M, Iobbi-Nivol C. 2016. Efficient respiration on TMAO requires TorD and TorE auxiliary proteins in Shewanella oneidensis. Res Microbiol 167:630–637. doi:10.1016/j.resmic.2016.05.004 27288570

[25] Conley BE, Intile PJ, Bond DR, Gralnick JA. 2018. Divergent Nrf family proteins and MtrCAB homologs facilitate extracellular electron transfer in Aeromonas hydrophila. Appl Environ Microbiol 84:1–14. doi:10.1128/AEM.02134-18 PMC6238070 30266730

[26] Williams KP, Gillespie JJ, Sobral BWS, Nordberg EK, Snyder EE, Shallom JM, Dickerman AW. 2010. Phylogeny of gammaproteobacteria. J Bacteriol 192:2305–2314. doi:10.1128/JB.01480-09 20207755 PMC2863478

[27] Augustus AM, Spicer LD. 2011. The MetJ regulon in gammaproteobacteria determined by comparative genomics methods. BMC Genomics 12:558. doi:10.1186/1471-2164-12-558 22082356 PMC3228920

[28] Urbanowski ML, Stauffer LT, Plamann LS, Stauffer GV. 1987. A new methionine locus, metR, that encodes a trans-acting protein required for activation of metE and metH in Escherichia coli and Salmonella typhimurium. J Bacteriol 169:1391–1397. doi:10.1128/jb.169.4.1391-1397.1987 3549685 PMC211958

[29] Raiford DW, Heizer EM, Miller RV, Akashi H, Raymer ML, Krane DE. 2008. Do amino acid biosynthetic costs constrain protein evolution in Saccharomyces cerevisiae? J Mol Evol 67:621–630. doi:10.1007/s00239-008-9162-9 18937004

[30] Ross DE, Brantley SL, Tien M. 2009. Kinetic characterization of OmcA and MtrC, terminal reductases involved in respiratory electron transfer for dissimilatory iron reduction in Shewanella oneidensis MR-1. Appl Environ Microbiol 75:5218–5226. doi:10.1128/AEM.00544-09 19542342 PMC2725455

[31] Sturm G, Richter K, Doetsch A, Heide H, Louro RO, Gescher J. 2015. A dynamic periplasmic electron transfer network enables respiratory flexibility beyond a thermodynamic regulatory regime. ISME J 9:1802–1811. doi:10.1038/ismej.2014.264 25635641 PMC4511935

[32] Barchinger SE, Pirbadian S, Sambles C, Baker CS, Leung KM, Burroughs NJ, El-Naggar MY, Golbeck JH. 2016. Regulation of gene expression in Shewanella oneidensis MR-1 during electron acceptor limitation and bacterial nanowire formation. Appl Environ Microbiol 82:5428–5443. doi:10.1128/AEM.01615-16 27342561 PMC4988178

[33] Bogard RW, Davies BW, Mekalanos JJ. 2012. MetR-regulated Vibrio cholerae metabolism is required for virulence. mBio 3:1–8. doi:10.1128/mBio.00236-12 PMC3448163 23015737

[34] Pan X, Sun C, Tang M, You J, Osire T, Zhao Y, Xu M, Zhang X, Shao M, Yang S, Yang T, Rao Z. 2020. LysR-type transcriptional regulator MetR controls prodigiosin production, methionine biosynthesis, cell motility, H _2_ O _2_ tolerance, heat tolerance, and exopolysaccharide synthesis in Serratia marcescens. Appl Environ Microbiol 86:e02241-19. doi:10.1128/AEM.02241-19 31791952 PMC6997736

[35] Yeung ATY, Torfs ECW, Jamshidi F, Bains M, Wiegand I, Hancock REW, Overhage J. 2009. Swarming of Pseudomonas aeruginosa is controlled by a broad spectrum of transcriptional regulators, including MetR. J Bacteriol 191:5592–5602. doi:10.1128/JB.00157-09 19592586 PMC2737960

[36] Miller J. 1972. Experiments in molecular genetics. Cold Spring Harbor Laboratory.

[37] Matsumoto A, Koga R, Kanaly RA, Kouzuma A, Watanabe K. 2021. Identification of a diguanylate cyclase that facilitates biofilm formation on electrodes by Shewanella oneidensis MR-1. Appl Environ Microbiol 87:e00201–21. doi:10.1128/AEM.00201-21 33637573 PMC8091010

[38] Saltikov CW, Newman DK. 2003. Genetic identification of a respiratory arsenate reductase. Proc Natl Acad Sci U S A 100:10983–10988. doi:10.1073/pnas.1834303100 12939408 PMC196913

[39] Kovach ME, Elzer PH, Steven Hill D, Robertson GT, Farris MA, Roop RM II, Peterson KM. 1995. Four new derivatives of the broad-host-range cloning vector pBBR1MCS, carrying different antibiotic-resistance cassettes. Gene 166:175–176. doi:10.1016/0378-1119(95)00584-1 8529885

[40] Kouzuma A, Oba H, Tajima N, Hashimoto K, Watanabe K. 2014. Electrochemical selection and characterization of a high current-generating Shewanella oneidensis mutant with altered cell-surface morphology and biofilm-related gene expression. BMC Microbiol 14:190. doi:10.1186/1471-2180-14-190 25028134 PMC4112983

[41] Kasai T, Kouzuma A, Watanabe K. 2018. CpdA is involved in amino acid metabolism in Shewanella oneidensis MR-1. Biosci Biotechnol Biochem 82:166–172. doi:10.1080/09168451.2017.1413326 29235426

[42] Kasai T, Tomioka Y, Kouzuma A, Watanabe K. 2019. Overexpression of the adenylate cyclase gene cyaC facilitates current generation by Shewanella oneidensis in bioelectrochemical systems. Bioelectrochemistry 129:100–105. doi:10.1016/j.bioelechem.2019.05.010 31153124

[43] Koga R, Matsumoto A, Kouzuma A, Watanabe K. 2020. Identification of an extracytoplasmic function sigma factor that facilitates c-type cytochrome maturation and current generation under electrolyte-flow conditions in Shewanella oneidensis MR-1. Environ Microbiol 22:3671–3684. doi:10.1111/1462-2920.15131 32548878

[44] Smyth GK. 2004. Linear models and empirical bayes methods for assessing differential expression in microarray experiments. Stat Appl Genet Mol Biol 3:Article3. doi:10.2202/1544-6115.1027 16646809

[45] Kouzuma A, Hashimoto K, Watanabe K. 2012. Roles of siderophore in manganese-oxide reduction by Shewanella oneidensis MR-1. FEMS Microbiol Lett 326:91–98. doi:10.1111/j.1574-6968.2011.02444.x 22092340

[46] Kouzuma A, Endoh T, Omori T, Nojiri H, Yamane H, Habe H. 2008. Transcription factors CysB and SfnR constitute the hierarchical regulatory system for the sulfate starvation response in Pseudomonas putida. J Bacteriol 190:4521–4531. doi:10.1128/JB.00217-08 18456803 PMC2446806

[47] Sperandio B, Gautier C, McGovern S, Ehrlich DS, Renault P, Martin-Verstraete I, Guédon E. 2007. Control of methionine synthesis and uptake by MetR and homocysteine in Streptococcus mutans. J Bacteriol 189:7032–7044. doi:10.1128/JB.00703-07 17675375 PMC2045202

[48] Klucar L, Stano M, Hajduk M. 2010. phiSITE: database of gene regulation in bacteriophages. Nucleic Acids Res 38:D366–70. doi:10.1093/nar/gkp911 19900969 PMC2808901

[49] Salgado H, Gama-Castro S, Lara P, Mejia-Almonte C, Alarcón-Carranza G, López-Almazo AG, Betancourt-Figueroa F, Peña-Loredo P, Alquicira-Hernández S, Ledezma-Tejeida D, Arizmendi-Zagal L, Mendez-Hernandez F, Diaz-Gomez AK, Ochoa-Praxedis E, Muñiz-Rascado LJ, García-Sotelo JS, Flores-Gallegos FA, Gómez L, Bonavides-Martínez C, Del Moral-Chávez VM, Hernández-Alvarez AJ, Santos-Zavaleta A, Capella-Gutierrez S, Gelpi JL, Collado-Vides J. 2024. RegulonDB v12.0: a comprehensive resource of transcriptional regulation in E. coli K-12. Nucleic Acids Res 52:D255–D264. doi:10.1093/nar/gkad1072 37971353 PMC10767902

[50] Turatsinze J-V, Thomas-Chollier M, Defrance M, van Helden J. 2008. Using RSAT to scan genome sequences for transcription factor binding sites and cis-regulatory modules. Nat Protoc 3:1578–1588. doi:10.1038/nprot.2008.97 18802439

[51] Crooks GE, Hon G, Chandonia JM, Brenner SE. 2004. WebLogo: a sequence logo generator. Genome Res 14:1188–1190. doi:10.1101/gr.849004 15173120 PMC419797

[52] Novichkov PS, Kazakov AE, Ravcheev DA, Leyn SA, Kovaleva GY, Sutormin RA, Kazanov MD, Riehl W, Arkin AP, Dubchak I, Rodionov DA. 2013. RegPrecise 3.0--a resource for genome-scale exploration of transcriptional regulation in bacteria. BMC Genomics 14:745. doi:10.1186/1471-2164-14-745 24175918 PMC3840689

