# Supplementary material for: Cross-regulation of amino acid synthesis and anaerobic electron transfer by MetR-mediated methionine signaling
Source: J Bacteriol. 2026 Jun 22;208(7):e00181-26. doi: 10.1128/jb.00181-26 (PMC13393422; doi:10.1128/jb.00181-26)
Supplement: Supplemental figures and tables — Figures S1 to S10, and Tables S1 and S2. [file jb.00181-26-s0009.pdf]

Supplementary Materials for

Cross-regulation of amino acid synthesis and anaerobic electron transfer by MetR-mediated methionine signaling

Hisae Mogi[1], Keisuke Tomita[1], Atsumi Hirose[1], Erika Yoshino[1], Takuya Kasai[1], Atsushi Kouzuma[1,2*], and Kazuya Watanabe[1]

*Corresponding author
Email: kouzuma@toyo.jp

This PDF file includes:

Figs. S1 to S10
Tables S1 to S2
References (1 to 3)

Other Supplementary Materials for this manuscript include the following:

Data S1 to S8

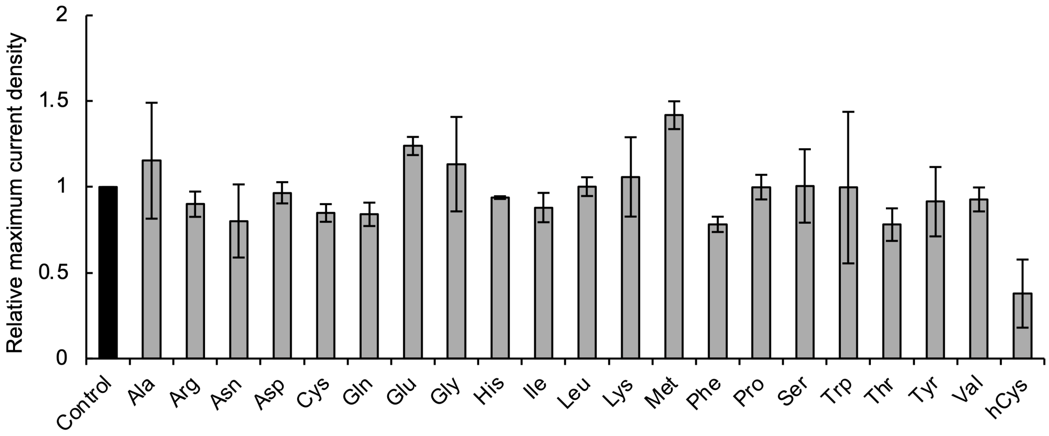

Fig. S1. Comparison of relative current generation by MR-1 in lactate-fed ECs supplemented with one of 20 amino acids and homocysteine (hCys). Each amino acid was added at a concentration of 130 μM. Bars and error bars represent means and standard deviations, respectively, calculated from the results of two independent experiments.

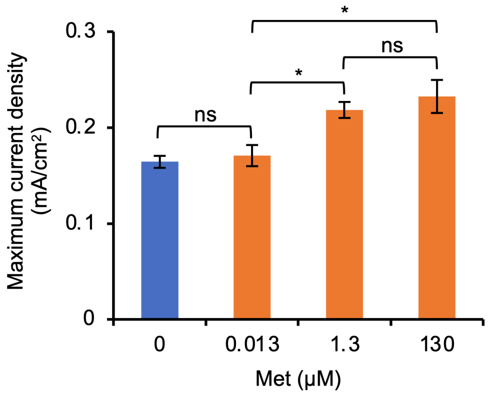

Fig. S2. Maximum current densities generated by MR-1 at different Met concentrations. Bars and error bars indicate means and standard deviations, respectively (n = 3 biological replicates). Asterisks indicate statistically significant differences ($P < 0.05$; one-way ANOVA followed by HSD test); ns, not significant.

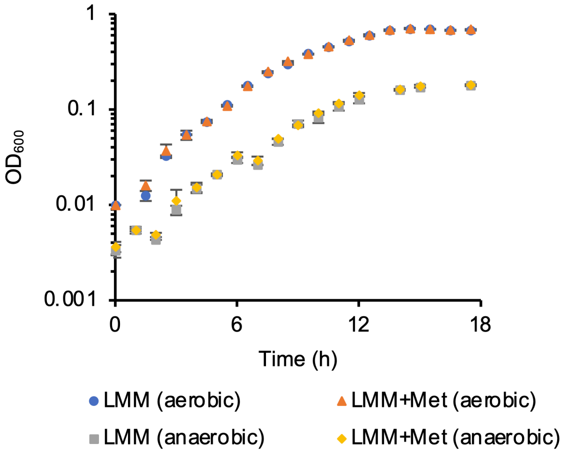

Fig. S3. Growth of MR-1 in LMM supplemented with or without 130 µM Met. Cells were cultured under aerobic or anaerobic (fumarate-reducing) conditions. Data points and error bars represent means and standard deviations, respectively, calculated from the results of two independent experiments.

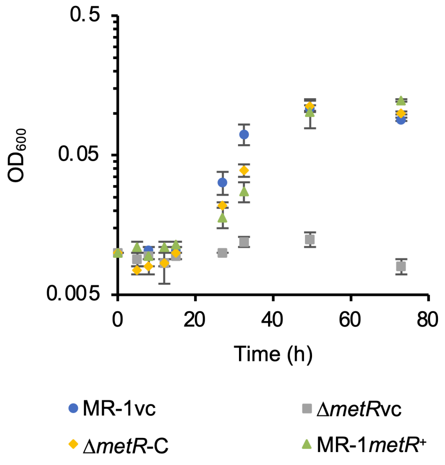

Fig. S4. Growth of MR-1vc, Δ*metR*vc, Δ*metR*-C, and MR-1*metR*$^{+}$ during anaerobic cultivation in LMM supplemented with TMAO as the electron acceptor. Data points and error bars represent means and standard deviations, respectively, calculated from the results of two independent experiments.

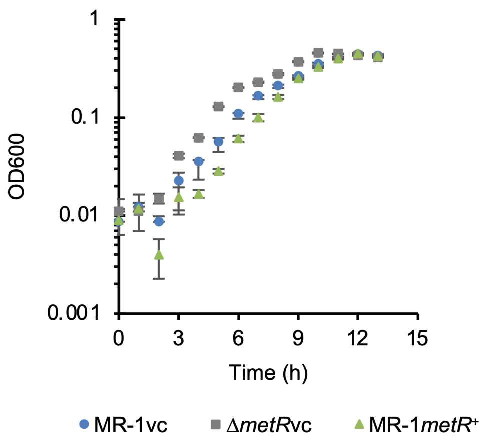

Fig. S5. Growth of Δ*metR*vc during anaerobic cultivation in LB supplemented with TMAO as the electron acceptor. For comparison, the growth curves of MR-1vc and MR-1*metR*$^{+}$ under the same culture conditions (shown in Fig. 2) are plotted. Data points and error bars represent means and standard deviations, respectively, calculated from the results of three independent experiments.

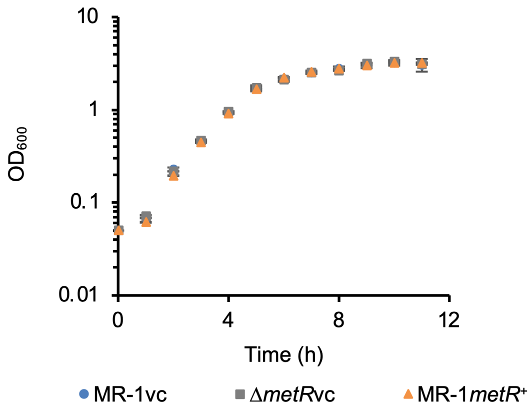

Fig. S6. Growth of MR-1vc, Δ*metR*vc, and MR-1*metR*$^+$ during aerobic cultivation in LB medium. Data points and error bars represent means and standard deviations, respectively, calculated from the results of two independent experiments.

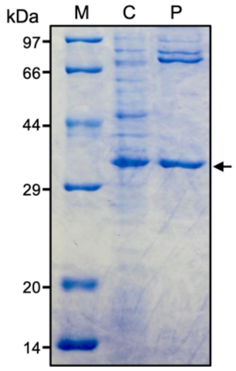

Fig. S7. SDS-PAGE of purified MetR. The arrow marks the band corresponding to MetR with a histidine tag at the C-terminal (33.4 kDa). Lane M, molecular weight marker; Lane C, *E. coli* BL21(DE3)(pET-metR) crude extract; and Lane P, purified extract.

AAAGAAATAGTCAGATCTCAATCACATTACCCGCTTA

CRP-binding site

AAGTGACTGATAAATACCAATGACTTAGTTAGCCTTA

–35 –10

CAGGT**G**GGATCTAATTCCACACATTAAATACGACTAA

+1

TGAGATTTGTTCTTATTTGATATGTGTTTAATGATG

MetR-binding site *omcA*

Fig. S8. Locations of the CRP- and MetR-binding sites upstream of *omcA*. The transcription start site (+1) of *omcA* is shown in bold, with the putative −35 and −10 promoter sequences underlined. The positions of CRP-binding and transcription start sites are based on other studies (1, 2).

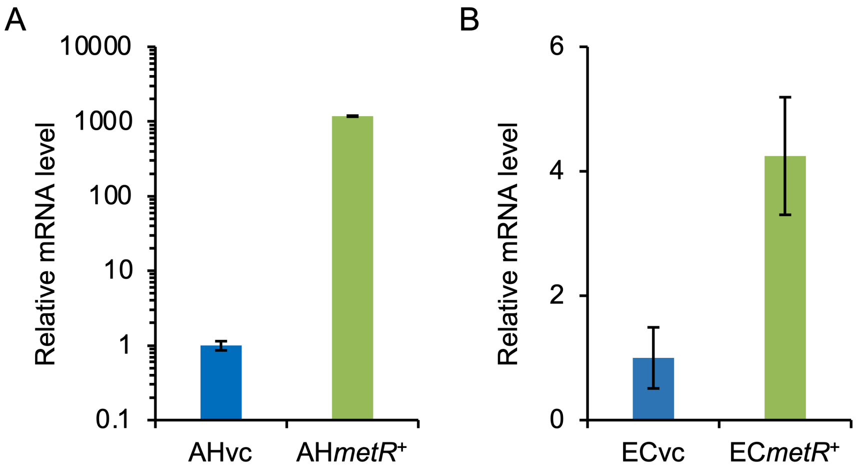

Fig. S9. Expression levels of *metR* in AH*metR*+ (A) and EC*metR*$^+$ (B) determined by quantitative RT-PCR. *A. hydrophila* strains were cultured aerobically in LMM. *E. coli* strains were cultured anaerobically in M9 glycerol medium supplemented with 0.5 mM IPTG to induce *metR* expression from the *lac* promoter carried on the expression vector pBBR1MCS-5. Results are presented as the relative levels of mRNA expression in the vector control strains (AHvc in panel A and ECvc in panel B). Bars and error bars represent means and standard deviations, respectively (n = 3 biological replicates).

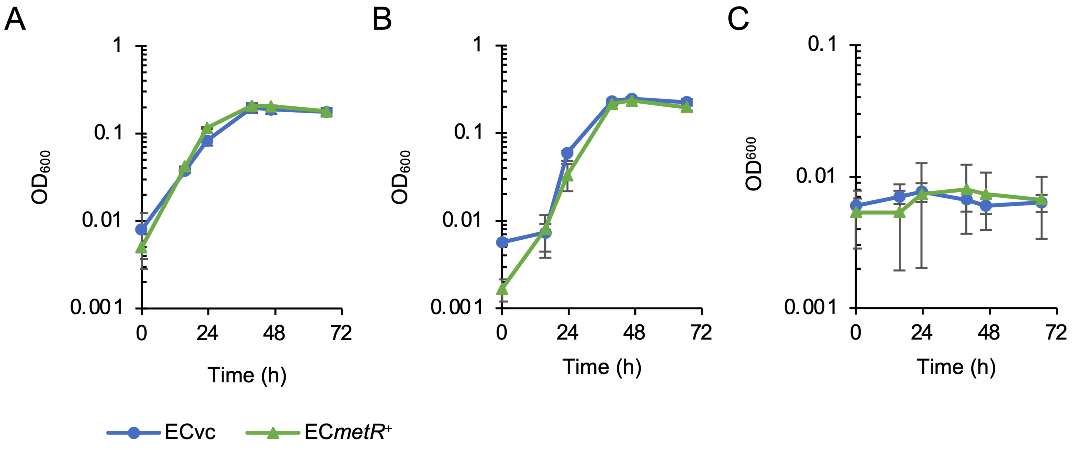

Fig. S10. Anaerobic growth of the ECvc and EC*metR*$^{+}$ strains. (A) Growth curves under fumarate-reducing conditions. (B) Growth curves under nitrate-reducing conditions. (C) Growth curves in the absence of electron acceptors as the negative control. In these experiments, cells were cultured in M9 glycerol medium to inhibit fermentative growth in the absence of electron acceptors. Data points and error bars represent means and standard deviations, respectively, calculated from the results of three independent experiments.

Table S1. Bacterial strains used in this study.

Strain or plasmid	Relevant characteristic	Source or reference
**Bacterial strains**		
Escherichia coli		
WM6026	Donor strain for conjugation; *lacI*^{q}, *rrnB3*, DE*lacZ4787*, *hsdR514*, DE(*araBAD*)*567*, *E*(*rhaBAD*)*568*, *rph-1*, *att-lambda*::*pAE12*-del(*oriR6K-cat*::*frt5*), DE(*endA*)::*frt*, *uidA*(*delMluI*)::*pir*(wt), *attHK*::pJK1006-*del1/2* (del*oriR6K-cat*::*frt5,* del*trfA*::*frt*)	William Metcalf, University of Illinois
DH5α	F^{-}, Φ80d*lacZ*ΔM15, Δ(*lacZYA-argF*)U169, *deoR*, recA*1*, *endA*1, *hsdR*17(r_K^-, m_K^+), *phoA*, *supE*44, λ^{-}, *thi*-1, *gyrA*96, *relA*1	Takara
ECvc	DH5α harboring pBBR1MCS-5, Gm^{r}	This study
EC*metR*$^{+}$	DH5α harboring pBBR-ECmetR, Gm^{r}	This study
Shewanella oneidensis		
MR-1	Wild type	ATCC
MR-1$_{vc}$	MR-1 harboring pBBR1MCS-5, Gm^{r}	This study
MR-1*metR*$^{+}$	MR-1 harboring pBBR-SOmetR, Gm^{r}	This study
Δ*metR*	SO_0817 (*metR*) disrupted	This study
Δ*metR*-C	Δ*metR* harboring pBBR-SOmetR, Gm^{r}	This study
Aeromonas hydrophila		
ATCC 7966	Wild type	ATCC
AHvc	ATCC 7966 harboring pBBR1MCS-5, Gm^{r}	This study
AH*metR*$^{+}$	ATCC 7966 harboring pBBR-AHmetR, Gm^{r}	This study
**Plasmids**		
pBBR1MCS-5	Broad-host-range vector, *lacZ* promoter, Gm^{r}	3
pBBR-SOmetR	pBBR1MCS-5-based plasmid expressing MR-1 *metR* (SO_0817)	This study
pBBR-AHmetR	pBBR1MCS-5-based plasmid expressing *A. hydrophila metR* (AHA_1955)	This study
pBBR-ECmetR	pBBR1MCS-5-based plasmid expressing *E. coli metR*	This study
pSMV10	9.1 kb mobilizable suicide vector; *oriR6K*, *mobRP4*, *sacB*, Km^{r}, Gm^{r}	Doug Lies, California Institute of Technology
pSMV-metR	pSMV10-based plasmid for MR-1 *metR* disruption	This study
pET-26b(+)	Expression vector, T7 promoter	Merck
pET-metR	pET-26b(+)-based plasmid expressing C-his-*metR*	This study

Table S2. Primers used in this study.

Primer	Sequence (5′–3′)	Modification, for use
metR-5-O-SpeI	GAAGGTAGACTAGTAATCCTGACCCACGGTTTTG	SpeI, *metR* disruption
metR-5-I	CTGACCAACAGCTGACACGGTTCGCAGATGTCTTAG	Linker, *metR* disruption
metR-3-I	GTGTCAGCTGTTGGTCAGCGCCAGCAGAAATCTAAC	Linker, *metR* disruption
metR-3-O-SpeI	GAAGGTAGACTAGTGAAGAGCAAGTAATTGGCCG	SpeI, *metR* disruption
SOmetR-F-EcoRI	CGGAATTCTGAGATAAGCCTATTCGG	EcoRI, pBBR-SOmetR construction
SOmetR-R-SpeI	GGACTAGTTAATCCGCTCTAAGAACTGC	SpeI, pBBR-SOmetR construction
AHmetR-F-EcoRI	CCGGAATTCGGCACAACAAGGAGGGATACC	EcoRI, pBBR-AHmetR construction
AHmetR-R-BamHI	CGCGGATCCTTACTGCGCCTTTACGTCGCC	BamHI, pBBR-AHmetR construction
ECmetR-F-EcoRI	CCGGAATTCCGAAGTGAAGGACTTTCATG	EcoRI, pBBR-ECmetR construction
ECmetR-R-PstI	AAAACTGCAGTTACAGGCGCGCTGGTGATCC	PstI, pBBR-ECmetR construction
qRT-omcA-F	GGATACGGCGTTGAAGATGT	qRT-PCR for *omcA* (SO_1779)
qRT-omcA-R	TGGTATCCGTTCCATTCCAT	qRT-PCR for *omcA* (SO_1779)
qRT-fccA-F	GTAATTATCGGCTCTGGTGGTG	qRT-PCR for *fccA* (SO_0970)
qRT-fccA-R	GCCTGTGGCTTAGTTTCTGC	qRT-PCR for *fccA* (SO_0970)
qRT-dmsB-F	GCGCCGTGTTTATGAATATGGC	qRT-PCR for *dsmB* (SO_1430)
qRT-dmsB-R	GCAGGCTTTAACACACACAGGC	qRT-PCR for *dsmB* (SO_1430)
qRT-metE-F	GAAGGTGTGGGCTTTACCAA	qRT-PCR for *metE* (SO_0818)
qRT-metE-R	AATCAACCGTCATGGCTTTC	qRT-PCR for *metE* (SO_0818)
qRT-16S-F	AGCGCAACCCCTATCCTTAT	qRT-PCR for MR-1 16S rRNA
qRT-16S-R	CGTAAGGGCCATGATGACTT	qRT-PCR for MR-1 16S rRNA
metR-F-NdeI	CATATGATCGAACTAAGACATCTGC	NdeI, pET-metR construction
metR-R-XhoI	CTCGAGGTTAGATTTCTGCTGGCGAG	XhoI, pET-metR construction
omcA-probe-F	CCACACATTAAATACGACTAATGAGATTTGTTCTTATTTG	*omcA* probe for EMSA
omcA-probe-R	CAAATAAGAACAAATCTCATTAGTCGTATTTAATGTGTGG	5'-Cy3, *omcA* probe for EMSA
omcAmu-probe-F	CCACACATTAAATACGACTAAGGGGATTTGGGGGTATTTG	*omcA*-m probe for EMSA
omcAmu-probe-R	CAAATACCCCCAAATCCCCTTAGTCGTATTTAATGTGTGG	5'-Cy3, *omcA*-m probe for EMSA
cymA-probe-F	GTATTAGCTGGAGTTGAAGTACTCTAACGCTCTGCTAAGC	*cymA* probe for EMSA
cymA-probe-R	GCTTAGCAGAGCGTTAGAGTACTTCAACTCCAGCTAATA	5'-Cy3, *cymA* probe for EMSA
glyA-probe-F	CTCAACTCGGCATTGAGGTGCATTCAAGTTAAAACTGTAG	*glyA* probe for EMSA
glyA-probe-R	CTACAGTTTTAACTTGAATGCACCTCAATGCCGAGTTGAG	5'-Cy3, *glyA* probe for EMSA
luxS-probe-F	GCATCTGACTGATATGAGATGATTTCATCTTAAGTTATC	*luxS* probe for EMSA
luxS-probe-R	GATAACTTAAGATGAAATCATCTCATATCAGTCAGATGC	5'-Cy3, *luxS* probe for EMSA
metR-probe-F	GATTTCAAGCGCAACATGAGCGAAATTCACTTGGAGTGATC	*metR* probe for EMSA
metR-probe-R	GATCACTCCAAGTGAATTTCGCTCATGTTGCGCTTGAAATC	5'-Cy3, *metR* probe for EMSA
dmsE-probe-F	CTTATGGTGTTTTTGAGAATGATTTTCTTTTATTTGATTTC	*dmsE* probe for EMSA
dmsE-probe-R	GAAATCAAATAAAAGAAAATCATTCTCAAAAACACCATAAG	5'-Cy3, *dmsE* probe for EMSA
qRT-EC16S-F	AGCGCAACCCTTATCCTTTG	qRT-PCR for *E. coli* 16S rRNA
qRT-EC16S-R	CGTAAGGGCCATGATGACTT	qRT-PCR for *E. coli* 16S rRNA
qRT-ECmetR-F	CGGCGAAAACGCGAATTACA	qRT-PCR for *E. coli metR*
qRT-ECmetR-R	GAAAATGCCGCCAGACATCC	qRT-PCR for *E. coli metR*
qRT-AH16S-F	TCGTGTCGTGAGATGTTGGG	qRT-PCR for *A. hydrophila* 16S
qRT-AH16S-R	GCAGTCTCCCTTGAGTTCCC	qRT-PCR for *A. hydrophila* 16S
qRT-AHmetR-F	TTGCCACAGTTGCATCCAGT	qRT-PCR for *A. hydrophila metR*
qRT-AHmetR-R	AGGGCGCTGATGGAATCAAA	qRT-PCR for *A. hydrophila metR*

Data S1. (separate file)
Upregulated genes in MR-1 cells grown under Met-supplemeted conditions.

Data S2. (separate file)
Downregulated genes in MR-1 cells grown under Met-supplemeted conditions.

Data S3. (separate file)
Upregulated genes in MR-1*metR*$^{+}$.

Data S4. (separate file)
Downregulated genes in MR-1*metR*$^{+}$.

Data S5. (separate file)
Putative MetR-bindig sites in the MR-1 genome.

Data S6. (separate file)
Predicted MetR regulon in MR-1.

Data S7. (separate file)
Putative CRP-binding sites in the MR-1 genome.

Data S8. (separate file)
Putative MetR-binding sites in the *A. hydrophila* genome.

References

1. Shao W, Price MN, Deutschbauer AM, Romine MF, Arkin AP. 2014. Conservation of transcription start sites within genes across a bacterial genus. mBio 5:e01398-14.
2. Kasai T, Kouzuma A, Nojiri H, Watanabe K. 2015. Transcriptional mechanisms for differential expression of outer membrane cytochrome genes *omcA* and *mtrC* in *Shewanella oneidensis* MR-1. BMC Microbiol 15:68.
3. Kovach ME, Elzer PH, Hill DS, Robertson GT, Farris MA, Roop RM, 2nd, Peterson KM. 1995. Four new derivatives of the broad-host-range cloning vector pBBR1MCS, carrying different antibiotic-resistance cassettes. Gene 166:175-176.
